# Supplementary material for: Perturbation of the mutated EGFR interactome identifies vulnerabilities and resistance mechanisms
Source: Mol Syst Biol. 2013 Nov 5;9:705. doi: 10.1038/msb.2013.61 (PMC4039310; doi:10.1038/msb.2013.61)

TABLE OF CONTENTS FOR SUPPLEMENTARY FILES

1. Supplementary Materials and Methods
    a. Supplementary Table S1
    b. Legends for Supplementary Figures
    c. Legends for Supplementary Data Files
2. Supplementary Figures 1 – 6

Title: Perturbation of the cancer-mutated EGFR interactome identifies vulnerabilities and resistance mechanisms

Authors: Jiannong Li, Keiryn Bennett, Alexey Stukalov, Bin Fang, Guolin Zhang, Takeshi Yoshida, Isamu Okamoto, Jae-Young Kim, Lanxi Song, Yun Bai, Xiaoning Qian, Bhupendra Rawal, Michael Schell, Florian Grebien, Georg Winter, Uwe Rix, Steven Eschrich, Jacques Colinge, John Koomen, Giulio Superti-Furga, and Eric B. Haura

SUPPLEMENTARY MATERIALS AND METHODS

Cell lines, plasmids, and reagents

Phoenix HEK293 cells were obtained from ATCC (Manassas, VA). 293FT cells were purchased from Invitrogen (Carlsbad, CA). Sources of lung cancer cell lines have been previously described (Haura et al, 2011; Li et al, 2010). Human immortalized airway epithelial cells (AALE) were provided by Drs. Heidi Greulich and William Hahn (Dana Farber) (Greulich et al, 2005). PC9GR cells were provided by Kinki University School (Osaka, Japan). HCC827ER and HCC4006ER cells were generated by our group. Briefly, HCC827 and HCC4006 cells with exon 19 deletion were continuously exposed to increasing doses of erlotinib (up to 4 µM) for 20 weeks, resulting in HCC827ER and HCC4006ER, which were resistant to erlotinib and confirmed by GLO cell proliferation assay (data not shown). HCC827 or HCC4006 stably expressing T790M or L858R EGFR mutant were generated by blasticidin selection for two weeks after EGFR T790M or L858R lentiviruses infection as previously described[2]. All cells were maintained in RPMI 1640 medium supplemented with 10% fetal bovine serum (FBS) from Invitrogen except for Phoenix and 293FT cells, which were cultured in DMEM medium with

10% FBS. cDNA for EGFR-Del was provided by Dr. William Pao (Vanderbilt University, Nashville, TN), while human ERBB3, ERBB2, CDC37, GRP78, and ERRFI1 were purchased from ORIGENE (Rockville, MD) or Addgene (Cambridge, MA). pfMSCV C-Strep-HA IRES GFP-GW, pfMSCV Strep-HA IRES GFP-GW, and pDONR201 vectors and retroviral plasmids containing N-HA tagged GRB2, STS1, SHC1, and AP2M1 were generously provided by Dr. Florian Grebien and Dr. Oliver Hantschel (CeMM, Vienna, Austria). cDNA for EGFR-Del, ERBB2, ERBB3, GRP78, ERRFI1, and CDC37 were amplified by PCR using the respective primers as shown in the Supplementary Data file S6. PCR products were inserted into pENTR D-TOPO vector for C-terminal fusion and pDONR201 vector for N-terminal fusion. The inserted constructs were then introduced into the respective destination vectors by Gateway LR Clonase^{TM} II Enzyme Mix Kit from Invitrogen as previously described (Li et al, 2010). Lentiviral shRNA targeting constructs in pLK0.1 targeting ARHG5 and CDC37, as well as non-targeting scrambled control, were purchased from OPEN Biosystems (Huntsville, AL); their sequences are shown in the Supplementary Data file S1. Erlotinib and BEZ235 were purchased from Chemie Tek (Indianapolis, IN), and dasatinib was provided by Bristol-Myers Squibb (New Brunswick, NJ).

Phosphoproteomics, Tandem affinity purification and data analysis

Phosphopeptide immunoprecipitation and purification were performed and analyzed by nano-LC-MS/MS as previously described (Li et al, 2010). A total of $1x10^8$ PC9 cells were treated with 1000 nM of erlotinib for 1 hour, with DMSO-treated cells assigned as the control. Tandem affinity purification (TAP) was performed as previously described (Haura et al, 2011). Strep-HA tagged baits expressed in PC9 or HCC827 or AALE cells were lysed in TNN-HS buffer (50 mM

HEPES pH 8.0, 150 mM NaCl, 5 mM EDTA, 0.5% NP-40, 50 mM NaF, 1.5 mM Na_3VO_4, 1.0 mM PMSF, and protease inhibitor cocktail). Insoluble material was removed by centrifugation at 39,443 × *g* for 15 min at 4°C. 200 µL StrepTactin sepharose (400 µL slurry/pulldown) was transferred to a 14-mL dust-free Falcon tube and washed with 2 × 1 mL TNN-HS buffer. The lysates (approximately 50 mg total protein from 5 × 15 cm plates) were added to the washed StrepTactin sepharose and rotated for 20 min at 4°C. The sepharose beads and supernatant were transferred to a spin column and gravity drained. The sepharose was washed with 4 × 1 mL TNN-HS buffer, and the bound proteins eluted with 3 × 300 µL freshly-prepared 2.5 mM D-biotin in TNN-HS buffer into a fresh dust-free 1.5 mL Eppendorf tube. 100 µL anti-HA agarose beads (200 µL slurry/pulldown) were transferred into a 1.5-mL Eppendorf tube, washed with 1 × 1 mL TNN-HS buffer, and centrifuged at 200 × *g* for 1 min at 4°C. The supernatant was removed and the agarose resuspended in 100 µL TNN-HS buffer. The anti-HA agarose beads were added to the biotin eluate and rotated for 1 h at 4°C. The samples were centrifuged at 200 × *g* for 1 min at 4°C and the supernatant removed. The agarose beads were resuspended in 1 mL TNN-HS buffer, the washed beads and buffer loaded into a fresh dust-free Biospin column and gravity drained. The anti-HA agarose was washed with 3 × 1 mL TNN-HS buffer and then with 2 × 1 mL TNN-HS buffer consisting of only HEPES, NaCl and EDTA. Retained proteins were eluted from the column directly into a glass HPLC vial with 500 µL 100 mM HCOOH and immediately neutralized with 125 µL 1 M TEAB. 200 µL were removed for immunoblot analysis as required. The remaining sample was frozen at -20°C until further processing. Proteins from the TEAB-neutralized acid eluate were reduced with dithiothreitol, alkylated with iodoacetamide and digested with trypsin. Multiples of 3% of the total eluate volume were desalted and concentrated with customized reversed-phase stage tips. The volume of the eluted

sample was reduced to approximately 2 μL in a vacuum centrifuge and reconstituted to 8 μL with 5% formic acid and multiples thereof. For TAP in AALE cells, the protocol was slightly modified. AALE cells were genetically tagged with each of baits, including GFP, EGFR WT, EGFR DEL, EGFR T790M separately. Ten 150-mm dishes of bait tagged AALE cells were used for single TAP experiment. Following elution from HA beads and neutralization with 1 M triethylammonium bicarbonate buffer (TEAB), each protein complex solution was lyophilized to completely dry in lyophilizer, resuspended with 35 μL Laemmli sample buffer, and desalted by short-time running SDS PAGE. Gels containing protein complex were cut out and conducted regular in-gel digestion process with trypsin. Resulting peptide mixture was submitted to LC MS/MS for further separation and identification. Two MS runs were performed for each sample.

Peak selection and conversion of RAW files into MGF format for subsequent protein identification were done by a combination of XCalibur (Thermo Scientific, Waltham, MA) and Trans Proteomics Pipeline(Keller et al, 2005) software tools. For the initial protein search, our group used Mascot (version 2.3.02, www.matrixscience.com) with 10 ppm parent and 0.6-Da fragment mass tolerance. Searches were limited to fully tryptic peptides with maximum of 1 missed cleavage, carbamidomethyl cysteine as fixed modification, and methionine oxidation as variable. Mascot peptide score threshold was set to 30, and at least 3 peptide identifications per protein were required. Searches were run against the human component of UniProtKB/SwissProt database (version 57), including all protein isoforms. The initial peptide identifications were used to deduce linear transformations for parent and fragment masses that would minimize the mean square deviation of measured masses from the theoretical ones. Calibrated peak files for TAP and phospho-enriched samples were searched against the same human proteins database by a combination of Mascot and Phenyx (version 2.5.14 by GeneBio, Switzerland) search engines.

Phosphorylations of serine, threonine, and tyrosine were included into the list of variable modifications for the analysis of phospho-enriched samples. The results of the two search engines were merged, requiring at least 2 distinct peptides with a score above threshold of either search engine. We also accepted single peptide hits, if the score was above a more stringent threshold (see Table S1). Spectra with conflicting peptide identifications were excluded from the combined result. Scores and mass tolerances were tuned to achieve 1% FDR of peptide identifications when searched against the reversed sequences database (see Table S1). The other parameters were the same as for the initial search.

Table S1

**Parameter**	**Value**
Parent mass tolerance, ppm	5.0 (TAP) / 3.0 (phospho)
Fragment mass tolerance, Dalton	0.3
Mascot score, ≥2 peptide hits	≥14
Mascot score, single peptide hit	≥28
Phenyx score, ≥2 peptide hits	≥4.2
Phenyx score, single peptide hit	≥4.75
Phenyx P value	≤0.001

For grouping of proteins based on shared peptides, identifications from all replicate MS runs were pooled together, and the proteins without protein-specific peptides were discarded. For dataset comparison and bait-prey interaction network construction, isoform information of the identified proteins was discarded. Pulldowns with tagged green fluorescent protein (GFP) were used as negative controls for other TAP pulldowns. Proteins identified in these pulldowns were considered as non-specific binders and removed from the resulting network. Each identified phosphorylation site was assigned a χ^2-based *P* value for the hypothesis that erlotinib treatment does affect the number of spectra for peptides that contain a given site in phosphorylated or unphosphorylated states:

$$p = P\left(x \geq \chi^2 \begin{pmatrix} N_{e^+,Ph^+} & N_{e^+,Ph^-} \\ N_{e^-,Ph^+} & N_{e^-,Ph^-} \end{pmatrix} \right),$$

where $N_{e+,Ph+}$ is the number of spectra for peptides containing a phosphorylated site in a sample treated with erlotinib. Significance threshold was set to $P = 0.1$. The significant phosphorylation sites of proteins that were also detected in TAP pulldowns were mapped directly to the protein-protein interaction network. For the other sites, the interactions of phosphorylated proteins with TAP proteins were searched using an internal database that aggregates public protein-protein interaction resources (IntAct, BioGrid, MINT, HPRD, and InnateDB). Phosphorylated proteins that were found to interact (be physically associated) with TAP proteins were added to the network together with their significant sites of phosphorylation.

Immunoprecipitation and protein expression analysis

Whole cell lysates were prepared using ice-cold TNN-HS buffer (50 mM HEPES, pH 8.0, 150 mM NaCl, 5 mM EDTA, 0.5% NP-40, 50 mM NaF, 1.5 mM Na_3VO_4, 1.0 mM PMSF, and protease inhibitor cocktail). Total cellular proteins were quantified using the Bio-Rad protein assay. For immunoprecipitation, 600 μg of total protein was incubated with 6 μL of mouse EGFR antibody from Cell Signaling (Beverly, MA) or mouse IgG from Santa Cruz Biotechnology (Santa Cruz, CA) as the control overnight at 4°C with rotation and then incubated with protein G-agarose beads (Roche, Indianapolis, IN) for 3 hours at 4°C. Beads were washed five times with TNN-HS buffer, and immunoprecipitated proteins were eluted from beads with 30 μL of 2X SDS-PAGE sample buffer from Bio-Rad (Hercules, CA) at 95°C for 5 minutes. Western blotting was performed as previously described (Li et al, 2010). Primary antibodies used in these studies consisted of EGFR, pTyr1068-EGFR, PARP, p44/42 MAPK, pThr202/Tyr204-p44/42 MAPK, AKT, pSer473-AKT, and tSRC from Cell Signaling; GRB2,

STAT3, and ERBB3 from Santa Cruz Biotechnology; HA, ERRFI1, and β-actin from Sigma (St. Louis, MO); SHC1 and CDC37 from Thermo Scientific (Rockford, IL); ERBB2 from Millipore (Billerica, MA); UBS3B from Rockland (Gilbertsville, PA); and IRDye™ 800CW-labeled goat-anti-rabbit and along with IRDye™ 680-labeled goat-anti-mouse secondary antibody from LI-COR (Lincoln, NE).

Cell viability assay

The cell viability assay (CellTiter-Glo) was conducted according to the recommendations for the CellTiter-Glo Luminescent Cell Viability Assay from Promega (Madison, WI). Cells were plated at 2-3 x 10^3 per well in black-wall 96-well plates from Thermo Fisher Scientific (Rochester, NY) and either transfected with siRNA or infected with 30 μL of lentiviruses plus 6 μg/mL polybrene (Sigma) for 5 days. Cell-Titer Glo reagent was added to each well, and luminescence was recorded using Victor plate reader from PerkinElmer (Walthom, MA).

Caspase 3 activity assays

Caspase 3 activity assays were performed according to the manufacturer's recommendations of PE-conjugated Monoclonal Active Caspase-3 Antibody Apoptosis Kit from BD Pharmingen (San Diego, CA). Briefly, $2x10^5$ cells were treated with 500 nM erlotinib, 1000 nM midostaurin, 500 nM of CEP-701, or combination with erlotinib at the same concentration for 24 hours, and then were resuspended in 0.5 ml of Cytofix/cytoperm solution for 20 min on ice. Pelleted cells were washed twice with Perm/Wash buffer and incubated with caspase-3 antibody for 30 min at room temperature. Cells were analyzed by flow cytometry (FACS 420, Becton Dickinson).

In-cell Western

PC9 cells were seeded at 8 x 10^3 per well in black-wall 96-well plates (Thermo Fisher Scientific). After transfection with siRNA library for 48 hours, cells were fixed with 150 μL/well of 3.7% formaldehyde/PBS for 20 minutes and then permeabilized by washing five times for 5 minutes with 200 μL of 0.1% Triton X-100/PBS; 50 μL of Odyssey Blocking Buffer (OBB) was added to each well for 90 minutes and then incubated with 50 μL of the primary antibody (pERK or pAKT) mixed with tERK (1:100 diluted in OBB) overnight at 4°C with gentle shaking. After five washes with 200 μL of 0.1% Tween 20-PBS, samples were incubated with IRDye™ 800CW-labeled goat-anti-rabbit and with IRDye™ 680-labeled goat-anti-mouse secondary antibody (1:800 dilution) in 0.2% Tween 20-OBB for 1 hour at room temperature without light. The plates were washed as before and then imaged using the LI-COR Odyssey Infrared Imaging Scanner in both 700- and 800-nm channels. The intensity of each dot was measured by Odyssey software and normalized to that of the tERK signaling. Background signals were evaluated in cells exposed to 10 μM of erlotinib or 10 μM of BEZ235 treatment for 6 hours for pERK and pAKT, respectively.

RNAi screen

The ON-TARGET plus Smart pool custom siRNA library included 102 siRNA pools containing four different siRNAs. ON-TARGET plus Non-Targeting Pool, Non-Targeting siRNA #4, and GAPD control siRNA pool were purchased from Thermo Scientific (Dharmacon); 4 pmol of each siRNA pool in a volume of 20 μL of 1x siRNA buffer from Dharmacon was delivered to each well in black-wall 96-well plates (NUNC, catalog no. 165305) using a Precision™ microplate liquid handler (BioTek). Each plate had triplicate wells for each siRNA pool, with six

wells for Non-Targeting siRNA pool as the control. The RNAi library information and daughter plate template are as shown in Supplemental Data file S3. For RNAi screen, 30 μL of OPTI-MEM containing 0.25 μL of lipofectamine RNAiMAX (Invitrogen) was then added to each well. After 5 minutes on the microplate shaker and 20 minutes of incubation at room temperature, 2 x 10^3 cells were delivered to the siRNA/liposome complexes in a total volume of 150 μL. The following day, we added the other 100 μL of medium containing 10% FBS to each well, and plates were then incubated at $37^oC/5\% CO_2$ for cell viability assay after 5 days of transfection and for pERK and pAKT in-cell Western after 48 hours of transfection. Data analysis was automated with RNAither package obtained from open-source Bioconductor website[4]. Viability and signaling change were determined for each target gene after normalization on ON-TARGET plus Non-Targeting pool control siRNA viability per plate. Hits were identified on the basis of statistical significance. For viability, significant hits were defined as (i) inhibition of cell viability >50% and (ii) *P* value <0.05 using Student's t-test. For pERK and pAKT in-cell Western, hits were ranked by Z-scores from per-plate normalization and target genes with absolute values of Z-scores more than 2 and *P* values less than 0.05 were considered statistically significant. To evaluate for reproducibility between two separate screen experiments in PC9 cells, Pearson correlation coefficients were used to assess pair-wise associations among measurements from different runs of experiments and averages within runs. Analyses were performed using SAS (version 9.2, SAS Institute Inc., Cary, NC), and a *P* value < 0.05 was considered statistically significant.

Quantitative RT-PCR analysis

RNA isolated from PC9 and PC9GR cells were infected with either ARHG5 shRNA or scrambled shRNA and cDNA transcription using RNeasy Mini kit and QuantiTect Reverse Transcription kit from Qiagen (Valencia, CA). Human ARHG5 probe and human 18s rRNA endogenous control were purchased from Applied Biosystems (Foster City, CA). Quantitative real-time PCR reactions were run in triplicate on MicroAmp® Optical 96-well reaction plates with barcode using the 7900HT Fast real-time PCR system, and data were analyzed with 2.3 SDS software and normalized by 18s endogenous control.

Chemical proteomics

c-Midostaurin was synthesized by derivatization of staurosporine with *N*-Boc-protected *m*-aminomethylbenzoic acid in the presence of HATU and diisopropylethylamine and subsequent deprotection with trifluoroacetic acid (all from Sigma-Aldrich). c-Midostaurin was immobilized on NHS-activated Sepharose 4 Fast Flow resin (GE Healthcare Bio-Sciences AB, Uppsala, Sweden) as described (Rix et al, 2007). Affinity chromatography was performed as reported previously with total cell lysates of PC9 and PC9GR cells containing each 5.5 mg of protein. Eluates were loaded on a SDS-PAGE gel, briefly run into the gel, and excised as a single piece before in-gel trypsin digestion and sample processing, which was done as described (Rix et al, 2007). LC-MS/MS analysis was performed in duplicate on a LTQ-Orbitrap MS (Thermo Fisher). The human UniProt database was appended by peptides corresponding to EGFR mutations known to be present in PC9 and PC9GR cells, and an EGFR inclusion list was defined before searching with the Mascot (Matrix Science) and Sequest (Thermo Fisher) search engines.

Kinase inhibition analysis

In vitro kinase assays were conducted using Merck Millipore's KinaseProfiler service according to the protocols detailed at http://www.millipore.com/techpublications/tech1/pf3036. Midostaurin was compared to DMSO for inhibition of the different alleles of EGFR including wild type, L858R, T790M, and T790M plus L858R at 0.0001, 0.0003, 0.001, 0.003, 0.01, 0.03, 0.1, 0.3, and 1 μM.

Human Phospho-kinase array assay

PC9 and PC9GR cells were infected with lentivirus expressing shRNA against ARHG5 or scrambled shRNA as control for 48 hours. The relative levels of phosphorylation of 46 kinase phosphorylation sites determination was performed using Human Phospho-Kinase Array Kit (R&D Systems, Minneapolis, MN). Briefly, two-hundred micrograms of total cell lysate protein were incubated with Phospho-Kinase array membranes overnight at 4°C. Detection of proteins was accomplished using the Detection Antibody Cocktail and enhanced chemiluminescence.

SUPPLEMENTARY FIGURE LEGENDS

Figure S1. EGFR network identified by tandem affinity purification (TAP) in lung cancer cells.

(A) Comparison of expression of exogenous tagged bait proteins with endogenous proteins in lung cancer cell lines. Lysates from PC9 or HCC827 cell lines stably expressing the indicated HA tagged bait protein and HA-tagged green fluorescent protein (GFP) as the control were immunoblotted with the relevant indicated antibodies. The equal protein loading was confirmed by β-actin evaluation.

(B) Comparison of EGFR complex (top panel) or ERBB3 complex (bottom panel) identified by TAP between PC9 and HCC827 cell lines. EGFR and ERBB3 TAP were performed in two lung cancer cell lines (PC9 and HCC827) stably expressing Strep-HA-tagged exon 19 deletion E746-A750 EGFR or tagged vision of ERBB3. The tagged GFP were expressed in both cell lines as the filter control. Overlapping proteins represent the preys pulled down by same baits in both PC9 and HCC827 cells; proteins listed in yellow or blue circles represent the preys pulled down by EGFR or ERBB3 only in PC9 or HCC827 cell lines, respectively.

(C) EGFR interaction validated by immunoprecipitation in PC9 cells. Lysates from PC9 cells were immunoprecipitated with mouse anti-EGFR antibody and mouse lgG antibody as the control, and immunoprecipitates were immunoblotted with indicated antibodies. WCE = whole cell extraction.

(D) Protein-protein interaction of EGFR, ERBB3 and ERBB2 identified by TAP in PC9 cells. TAP experiments were performed in PC9 cells stably expression Strep-HA-tagged exon 19 deletion E746-A750 EGFR, ERBB3 and ERBB2 and tagged version of GFP as the control. Prey proteins (yellow ellipse) were identified by TAP using EGFR, ERBB3, and ERBB2 as the bait

proteins (blue rectangle) following subtraction of non-specific proteins using control GFP as bait in PC9 cells. The line between proteins indicates bait-prey relationship. The protein-protein interaction map is visualized by Cytoscape software (http://cytoscape.org).

Figure S2. RNAi screen reproducibility analysis. Total 102 siRNA pools were respectively delivered to each well in 96-well plates by a Precision^{TM} microplate liquid handler and two individual RNAi screens for cell viability assay using Celltiter Glo were performed in PC9 cells. Viability changes were determined for each target gene after normalization on ON-TARGET plus Non-Targeting pool control siRNA. The reproducibility between two separate screen experiments was evaluated by Pearson correlation coefficients using SAS (version 9.2, SAS Institute Inc., Cary, NC), and a P value < 0.05 was considered statistically significant. Pearson correlation coefficients (**A**) and Scatter plot matrix (**B**) within each experiment, as well as across experiments. In A, r represents Pearson correlation coefficient and p represents P value. Total sample size is N = 102. There are triplicate wells for each gene in experiment 1 and duplicate wells for each gene in experiment 2.

Figure S3. Cell viability hits in PC9, HCC827, and HCC4006 cell lines identified by RNAi. Total 102 siRNA pools were respectively delivered in triplicate to each well in 96-well plates by a Precision^{TM} microplate liquid handler and Non-Targeting pool siRNA as the control. Cells were transfected by RNAi library with the final concentration of 20 nM of each siRNA using lipofectamine RNAiMAX reverse transfection procedure as described in detail in Materials and Methods, and cell viability was performed by CellTiter Glo after 5-day transfection. The significant hits were defined as 1) the cell viability inhibition is more than 50% compared with

ON-TARGET plus non-targeting pool control siRNA and 2) *P* value < 0.05 using Student's t-test. Results are means ± SD for triplicate data points.

Figure S4. Transfection efficiency in the different cell lines. Cells were seeded in duplicate in 60-mm dishes and transfected with 20 nM of siGLO using lipofectamine^{TM} RNAiMAX reverse transfection procedure for 72 hours. Detection of GFP-positive population cells was performed by Flow cytometry. Results for GFP percentage for each cell line are means ± SD for duplicate data points.

Figure S5. Signaling studies in ARHG5 knockdown cells. PC9 and PC9GR cells were infected with two individual lentivirus expressing shRNA against ARHG5 and scrambled shRNA as control. **(A)** Lysates from both cells after 72 hours of infection were transferred to membranes and signaling changes were evaluated with indicated antibodies by Western blotting. **(B)** The total cell lysate protein from both cell lines after 48-hour infection were incubated with Phospho-kinase array membranes, and relative levels of phosphorylation of 46 kinase phosphorylation sites were determined using the detection antibody cocktail and enhanced chemiluminescence according to the recommendation of Human Phospho-Kinase Array Kit

Figure S6. Contributions of TAP and phosphoproteomic data in interactome and target identification. The number of proteins identified by tandem affinity purification (TAP) and phosphotyrosine proteomics (pY) are shown in the Venn diagram and indicate their contribution to interactome, function analysis, and core network.

SUPPLEMENTARY DATA FILES

Data File S1. Proteins identified by TAP approach using different baits in PC9, HCC827, and AALE cell lines. All proteins were identified by TAP approach in PC9 and HCC827 cell lines using different baits, with GFP bait as the negative control. Each table is named by cell line – bait and shows the single TAP results. Proteins are sorted alphabetically, and each protein contains UniProt ID, name, description, and relevent parameters. Peptide counts (pc) describe the number of unique peptides identified for each protein. Spectral counts (sc) describe the number of spectra for these unique peptides of each identified protein. Sequence coverages (SC) are based on these unique peptides and the amino acid sequences of the respective proteins. SUP indicates superspecific peptides only, and SP indicates specific peptides only. For AALE TAP datasheet, the experiments were performed with total cell lysates of AALE cells stably expressing EGFR wild type, EGFR-del, and GFP as the control bait as described in Materials and Methods. Database searches of MS data from one biological sample and duplicate technique MS runs investigated were performed using Mascot and Sequest and summarized using Scaffold. The protein report worksheet was exported from Scaffold after the data were filtered with the following criteria: minimal 50% on protein probability, minimal 1 peptides per protein, and minimal 80% and 10 PPM on peptide probability.

Data File S2. Phosphorylation sites identified by phosphoproteomics approach in PC9 cells treated with or without erlotinib. PC9 cells were treated with 1 µM of erlotinib for 1 hour, with DMSO-treated cells as the control. Phospho-sites of each protein were identified by the phosphoproteomics approach as described in Materials and Methods. Each number represents the

total spectral counts across the 2 biological samples and 2 technical runs of each sample. “SC_PHOS_” and “SC_” indicate the number of spectra for peptides in a given site in phosphorylated and unphosphorylated states, respectively. *P* value is calculated based on 2 x 2 contingency table using the formula shown in Supplementary Materials and Methods.

Data File S3. Functional analysis by custom RNAi library in EGFR-mutant non-small cell lung cancer cell lines. “RNAi library information” lists the 102 genes and relative information, including the sequence of 4 individual siRNA for each gene. “RNAi daughter plate template” indicates the distribution of each siRNA with triplicate in 96-well plates; each plate had six wells of On-TARGET plus Non-Targeting smart pool siRNA as the control and total seven plates per set cover 102 siRNA. To avoid the edge effect, all edge wells were blank. RNAi results, including the cell viability results detected by CellTiter Glo after 5-day transfection in PC9 and PC9GR, HCC827 and HCC827ER, and HCC4006 and HCC4006ER cell lines are also shown. After normalization on ON-TARGET plus Non-Targeting pool control siRNA per plate, cell viability, standard deviation, and *P* value for each target gene were automatically calculated with RNAither. “15 gene screen in 11 cell lines” indicated the average of cell viability and standard deviation from the three technique replicates. The cells were transfected with RNAi and cell viability was detected as described above.

Data File S4. Drug list derived from the core EGFR network. Drugs interacting with the 14 core proteins plus CDC37 were searched from 4 drug data sources as described in Materials and Methods. All drugs listed in the table are sorted alphabetically, with targeted protein and drug source shown for each.

Data File S5. Raw data of midostaurin affinity chromatography experiments. Midostaurin affinity chromatography experiments were performed with total cell lysates of PC9 and PC9GR cells as described in Materials and Methods. Database searches of MS data from two individual experiments investigated were performed using Mascot and Sequest and summarized using Scaffold. The peptide report worksheet was exported from Scaffold after the data were filtered with the following criteria: minimal 50% on protein probability, minimal 2 peptides per protein, and minimal 95% on peptide probability.

Data File S6. Sequence of primers for PCR and shRNA for ARHG5 and CDC37. "PCR-primer" lists six baits that we generated for TAP and their relevant protein name, UniProt ID, HA tagged C- or N-terminal amino acid, and the sequence of forward and reverse primers for cDNA amplification. "shRNA Seq" lists the sequence of each lentiviral shRNA against either ARHG5 or CDC37.

REFERENCES

Greulich H, Chen TH, Feng W, Janne PA, Alvarez JV, Zappaterra M, Bulmer SE, Frank DA, Hahn WC, Sellers WR, Meyerson M (2005) Oncogenic Transformation by Inhibitor-Sensitive and -Resistant EGFR Mutants. *PLoS Med* **2:** e313

Haura EB, Muller A, Breitwieser FP, Li J, Grebien F, Colinge J, Bennett KL (2011) Using iTRAQ combined with tandem affinity purification to enhance low-abundance proteins associated with somatically mutated EGFR core complexes in lung cancer. *J Proteome Res* **10:** 182-190

Keller A, Eng J, Zhang N, Li XJ, Aebersold R (2005) A uniform proteomics MS/MS analysis platform utilizing open XML file formats. *Mol Syst Biol* **1:** 2005 0017

Li J, Rix U, Fang B, Bai Y, Edwards A, Colinge J, Bennett KL, Gao J, Song L, Eschrich S, Superti-Furga G, Koomen J, Haura EB (2010) A chemical and phosphoproteomic characterization of dasatinib action in lung cancer. *Nat Chem Biol* **6:** 291-299

Rix U, Hantschel O, Durnberger G, Remsing Rix LL, Planyavsky M, Fernbach NV, Kaupe I, Bennett KL, Valent P, Colinge J, Kocher T, Superti-Furga G (2007) Chemical proteomic profiles of the BCR-ABL inhibitors imatinib, nilotinib, and dasatinib reveal novel kinase and nonkinase targets. *Blood* **110:** 4055-4063

Supp Figure 1A

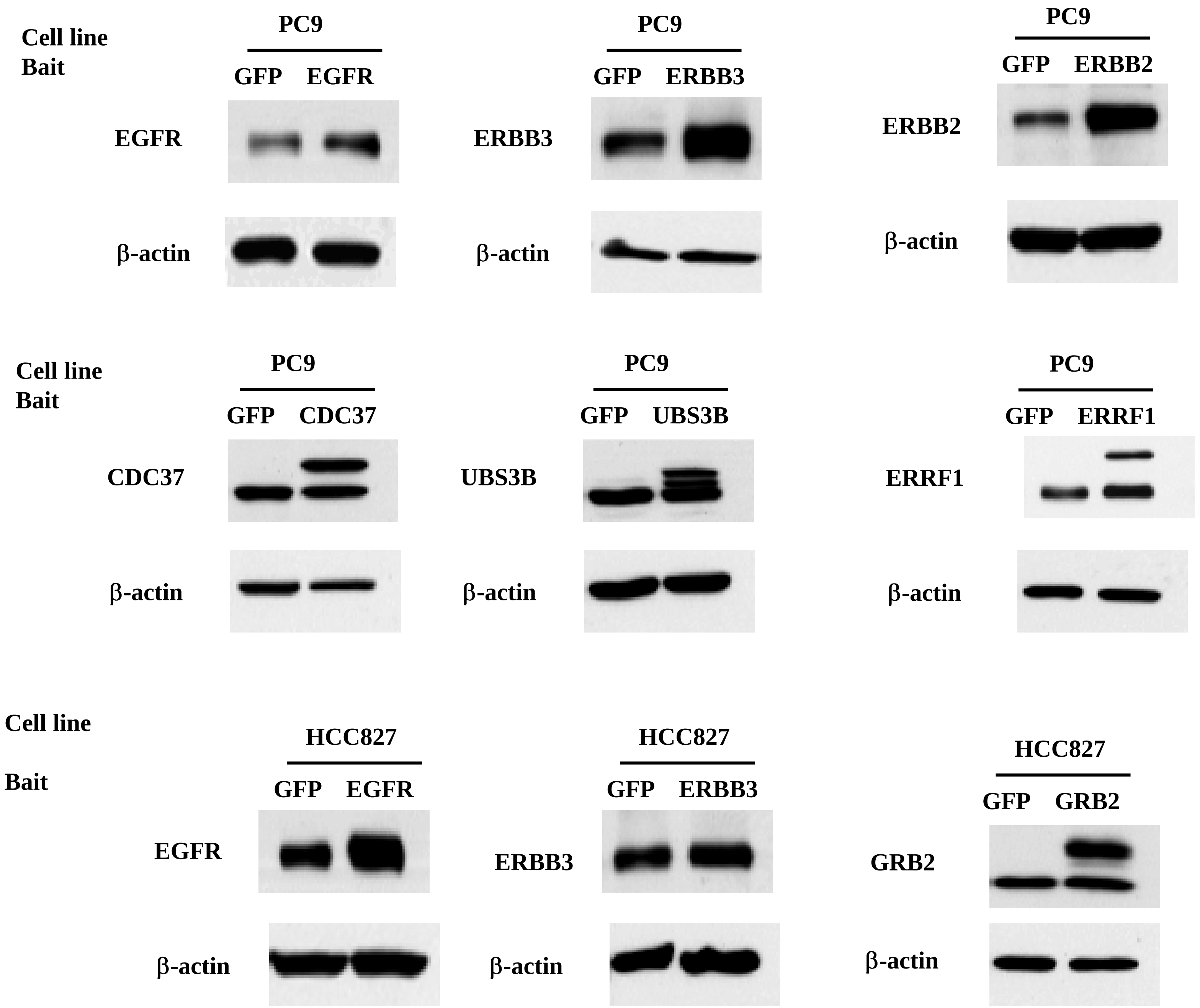

Supp Figure 1B

EGFR complex

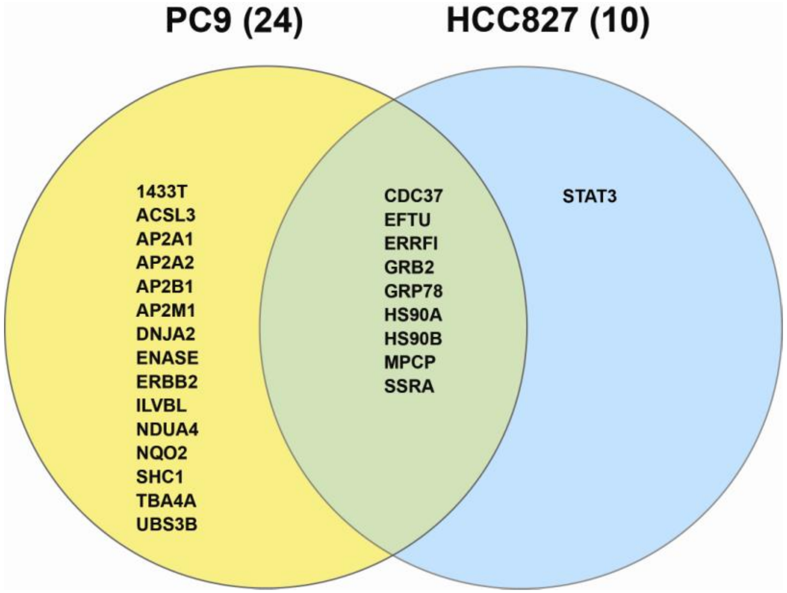

ERBB3 complex

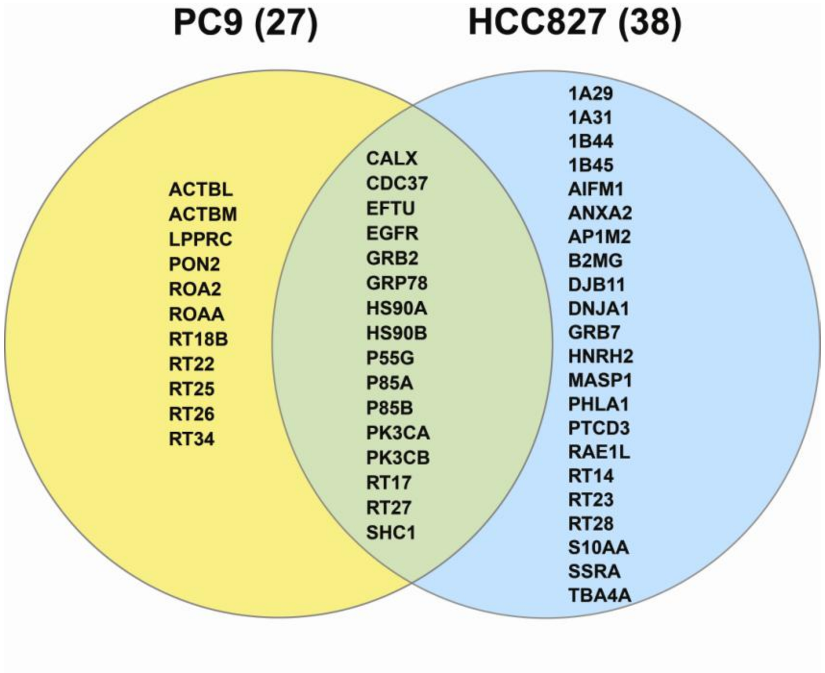

Supp Figure 1C

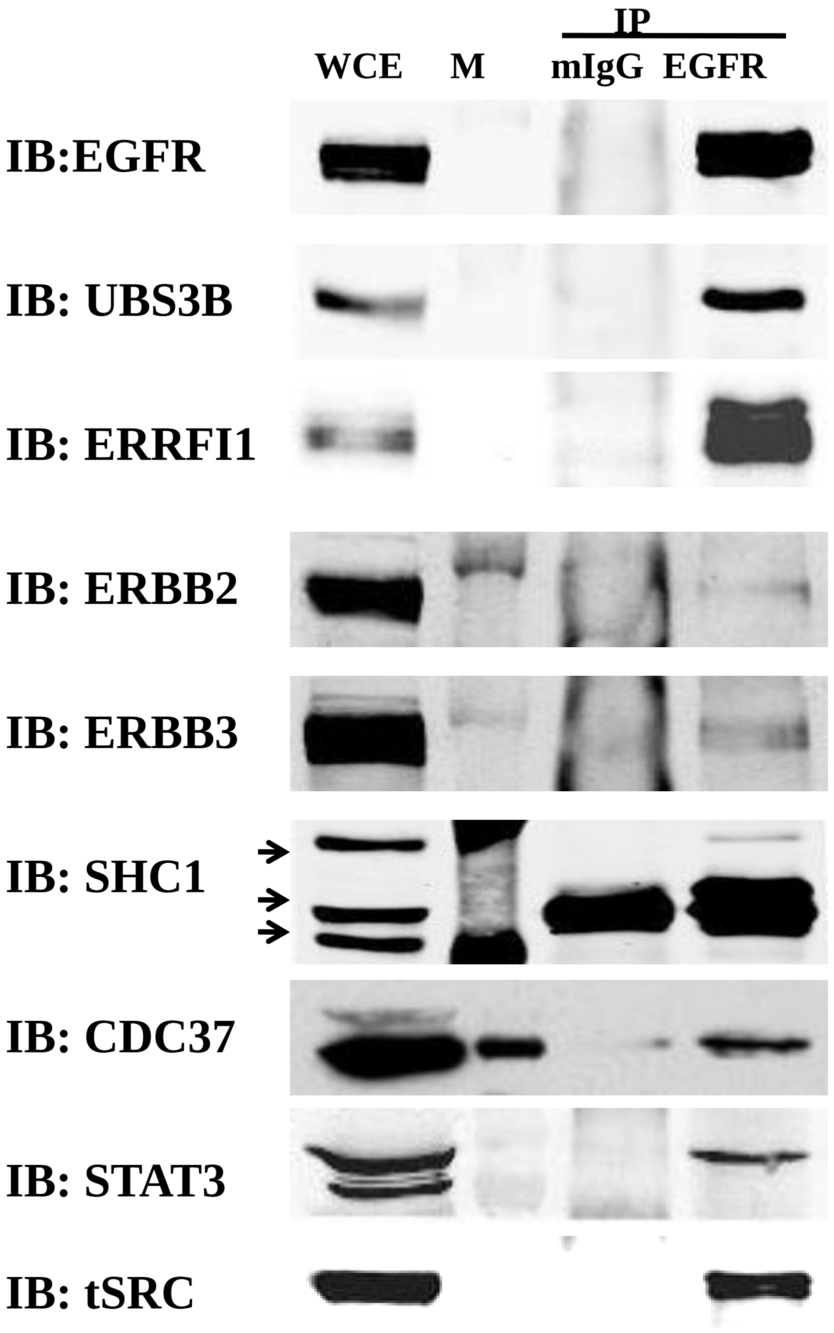

Supp Figure 1D

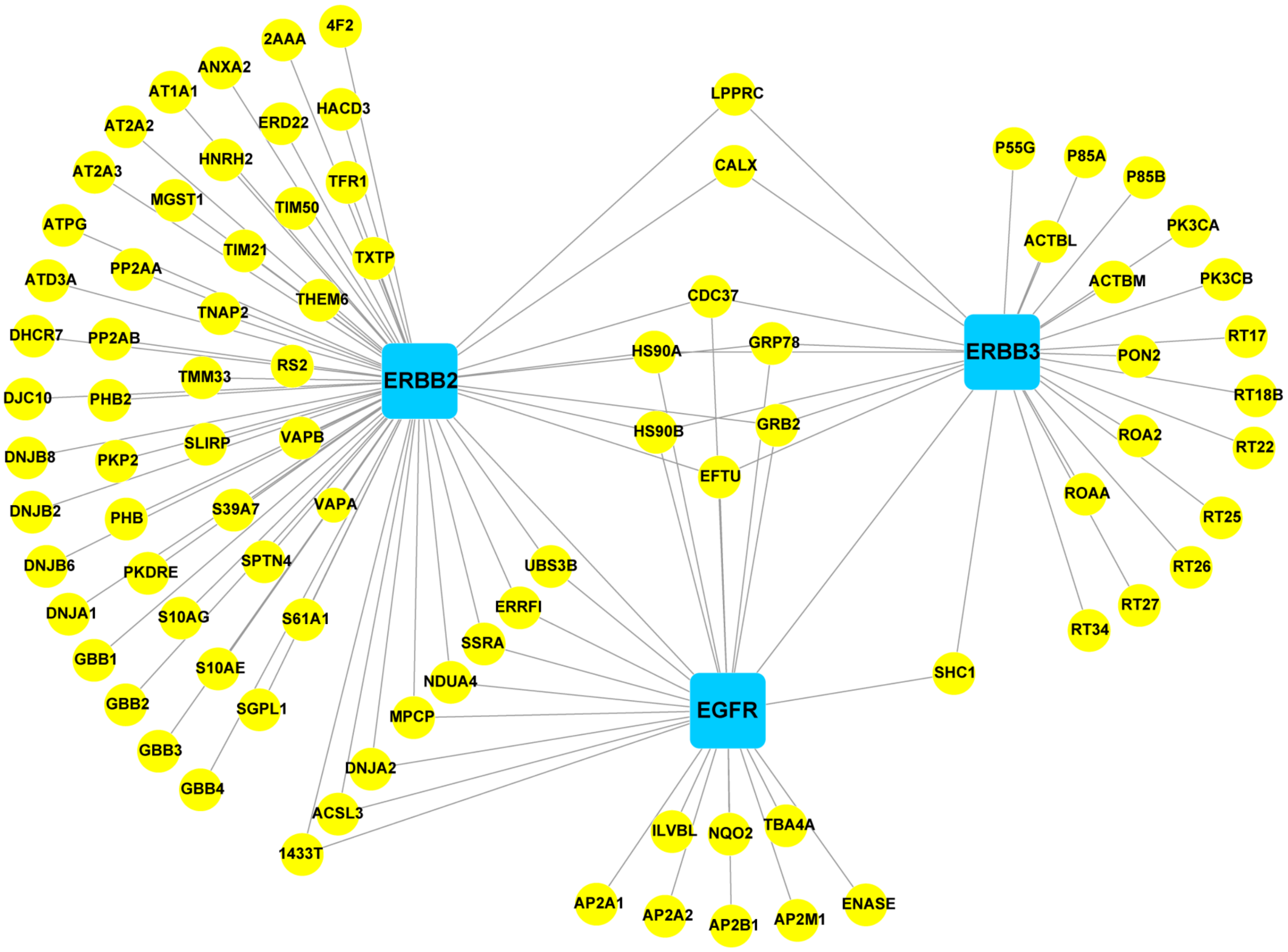

Supp Figure 2A

	Well 1-Exp1	Well 2-Exp1	Well 3-Exp1	AVE-Exp1	Well 1-Exp2	Well 2-Exp2	AVE-Exp2
**Well 1-Exp1**	r =1	r = 0.947<br>p < .0001	r = 0.948<br>p < .0001	r = 0.984<br>p < .0001	r = 0.952<br>p < .0001	r = 0.946<br>p < .0001	r = 0.955<br>p < .0001
**Well 2-Exp1**		r =1	r = 0.937<br>p < .0001	r = 0.980<br>p < .0001	r = 0.944<br>p < .0001	r = 0.937<br>p < .0001	r = 0.947<br>p < .0001
**Well 3-Exp1**			r =1	r = 0.980<br>p < .0001	r = 0.941<br>p < .0001	r = 0.943<br>p < .0001	r = 0.948<br>p < .0001
**AVE-Exp1**				r =1	r = 0.964<br>p < .0001	r = 0.960<br>p < .0001	r = 0.968<br>p < .0001
**Well 1-Exp2**					r =1	r = 0.974<br>p < .0001	r = 0.994<br>p < .0001
**Well 2-Exp2**						r =1	r = 0.993<br>p < .0001
**AVE-Exp2**							r =1

Supp Figure 2B

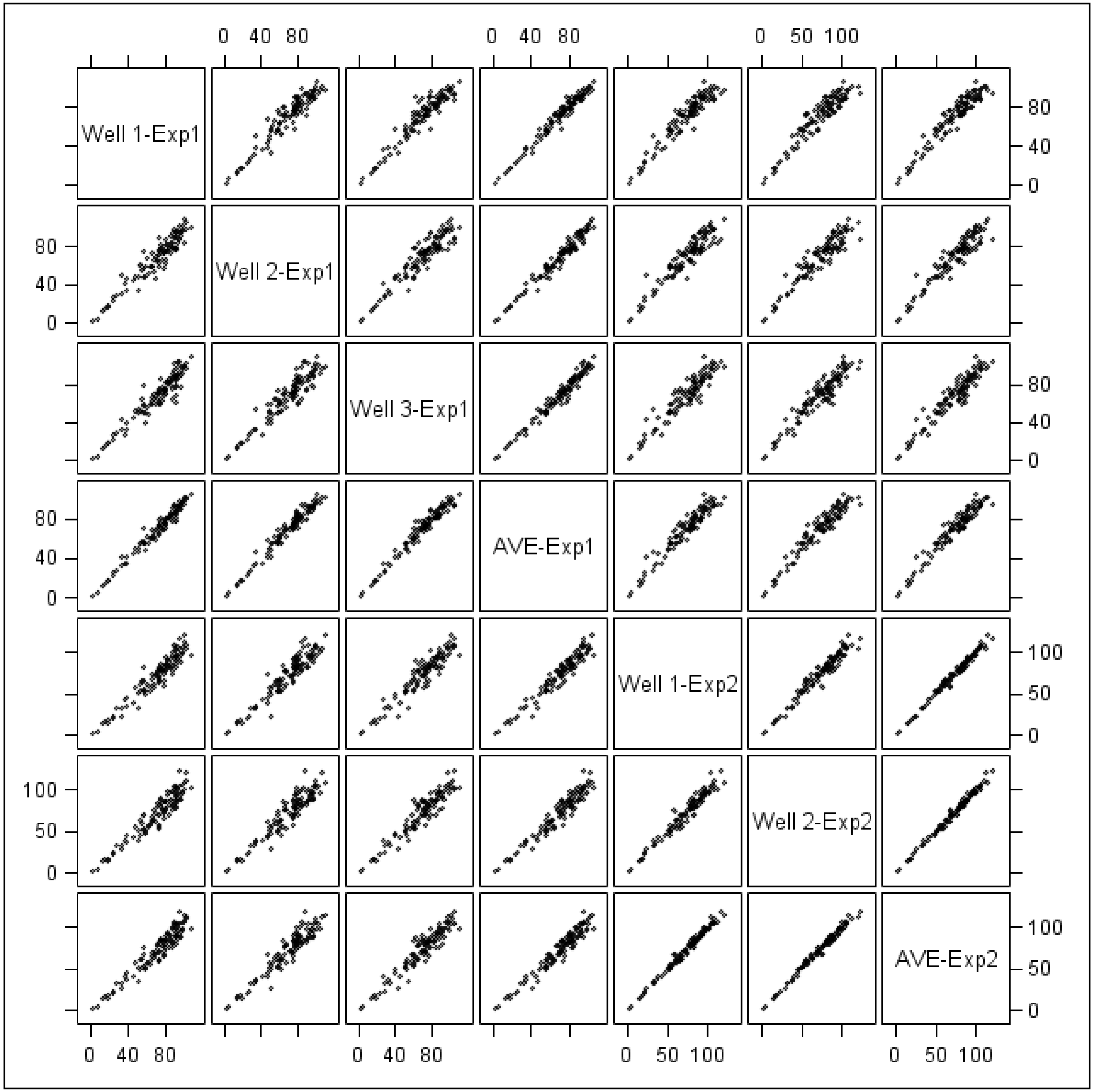

Supp Figure 3

PC9		HCC827		HCC4006	
**Hits**	**Viability (%)**	**Hits**	**Viability (%)**	**Hits**	**Viability (%)**
PK3CA	5.0 ± 1.3	HS90B	8.4 ± 3.0	HS90B	3.2 ± 0.5
P85B	13.2 ± 0.5	CDK9	15.1 ± 0.6	AAK1	3.8 ± 0.8
GRB2	14.2 ± 0.6	CD11A	16.2 ± 0.4	CD11B	5.2 ± 1.4
GPR78	17.3 ± 0.4	PK3CA	16.9 ± 1.7	CTNB1	7.3 ± 1.4
CD11A	18.1 ± 0.2	GPR78	18.5 ± 1.6	ERBB4	12.5 ± 10.2
ARHG5	25.2 ± 0.8	P85B	19.8 ± 0.7	EGFR	14.3 ± 0.7
CD11B	26.0 ± 1.9	CD11B	21.7 ± 5.8	SHC1	14.6 ± 5.3
EGFR	34.6 ± 1.0	CTNB1	24.9 ± 4.3	CDK13	16.8 ± 5.2
SHC1	34.9 ± 7.6	ARAF	27.5 ± 4.4	CDK9	17.2 ± 1.2
MK12	35.3 ± 3.1	MK12	28.6 ± 0.7	P85B	18.2 ± 0.7
GLU2B	40.5 ± 3.9	GLU2B	30.7 ± 2.3	CD11A	19.3 ± 1.9
ARAF	42.1 ± 8.5	AAK1	31.6 ± 2.4	GPR78	22.8 ± 1.5
CDK9	44.3 ± 0.7	GRB2	32.8 ± 2.9	GRB2	23.0 ± 1.7
P55G	49.1 ± 1.0	EGFR	33.1 ± 4.1	PK3CB	26.1 ± 13.4
AT2A2	49.7 ± 9.0	SHC1	35.4 ± 1.7	PK3CA	27.8 ± 0.5
KS6A3	49.9 ± 12.1	CDK1	37.2 ± 0.6	MK12	27.9 ± 1.6
		YES	41.8 ± 2.0	GLU2B	29.7 ± 1.7
		FGFR4	42.3 ± 0.9	ARAF	30.9 ± 0.9
		PKP2	43.4 ± 1.8	PLEC	36.0 ± 10.6
		P55G	43.9 ± 2.4	CDK1	36.5 ± 1.0
		ARHG5	45.4 ± 4.1	KS6A4	36.7 ± 7.4
		ERBB4	46.9 ± 9.1	FGFR4	38.4 ± 2.1
		KS6A4	48.8 ± 1.8	KS6A1	40.6 ± 2.6
		ICK	48.9 ± 2.9	BLK	40.7 ± 11.5
		DHCR7	49.7 ± 5.8	YES	40.9 ± 3.4
				P55G	41.5 ± 0.4
				PKP2	42.3 ± 1.3
				MYH9	43.7 ± 3.1
				STAT3	46.2 ± 3.2
				CALX	47.4 ± 1.8
				ARHG5	47.6 ± 1.0
				ICK	48.8 ± 1.0
				PRKDC	49.6 ± 4.4

Supp Figure 4

**Cell lines**	**GFP + (%)**
PC9	97.1 ± 0.6
PC9GR	99.1 ± 0.1
HCC827	98.3 ± 0.1
HCC827T790M	91.3 ± 1.1
HCC4006	98.2 ± 0.2
HCC827ER	94.8 ± 0.1
HCC4006ER	97.9 ± 0.8
H460	84.8 ± 0.8
A549	95.6 ± 0.2
H23	95.8 ± 1.1
H157	97.3 ± 0.0
H441	87.1 ± 0.4
H1437	78.5 ± 2.1
H1299	97.6 ± 0.9
H520	94.5 ± 0.6
H1703	96.6 ± 0.2
H2170	94.3 ± 1.1

Supp Figure 5A

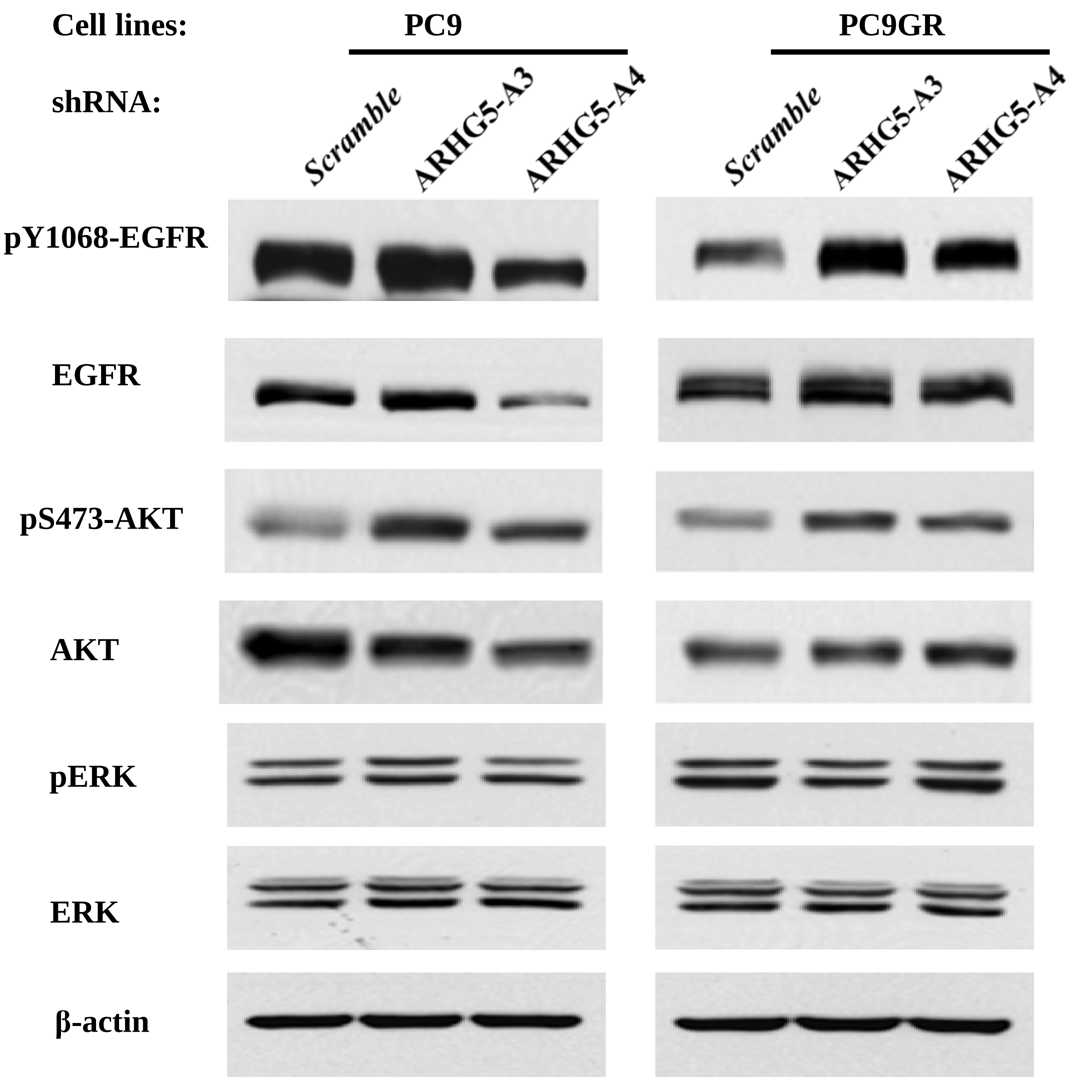

Supp Figure 5B

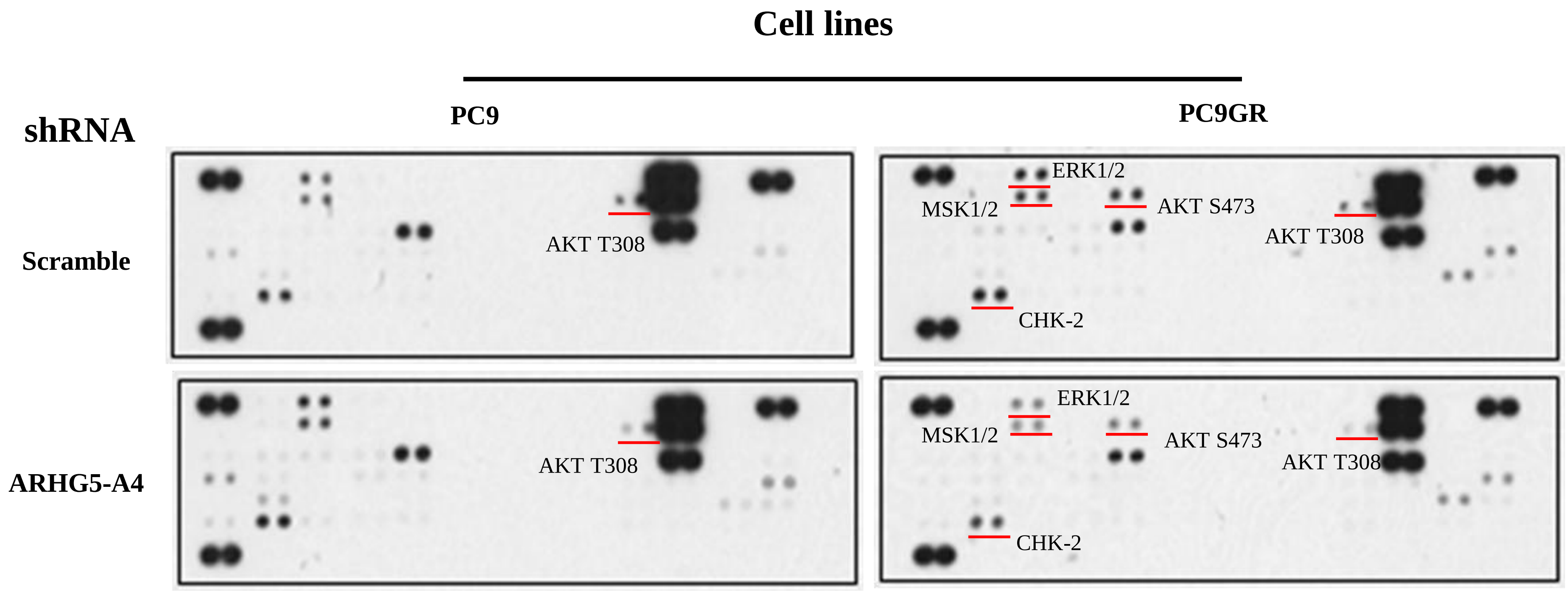

Supp Figure 6

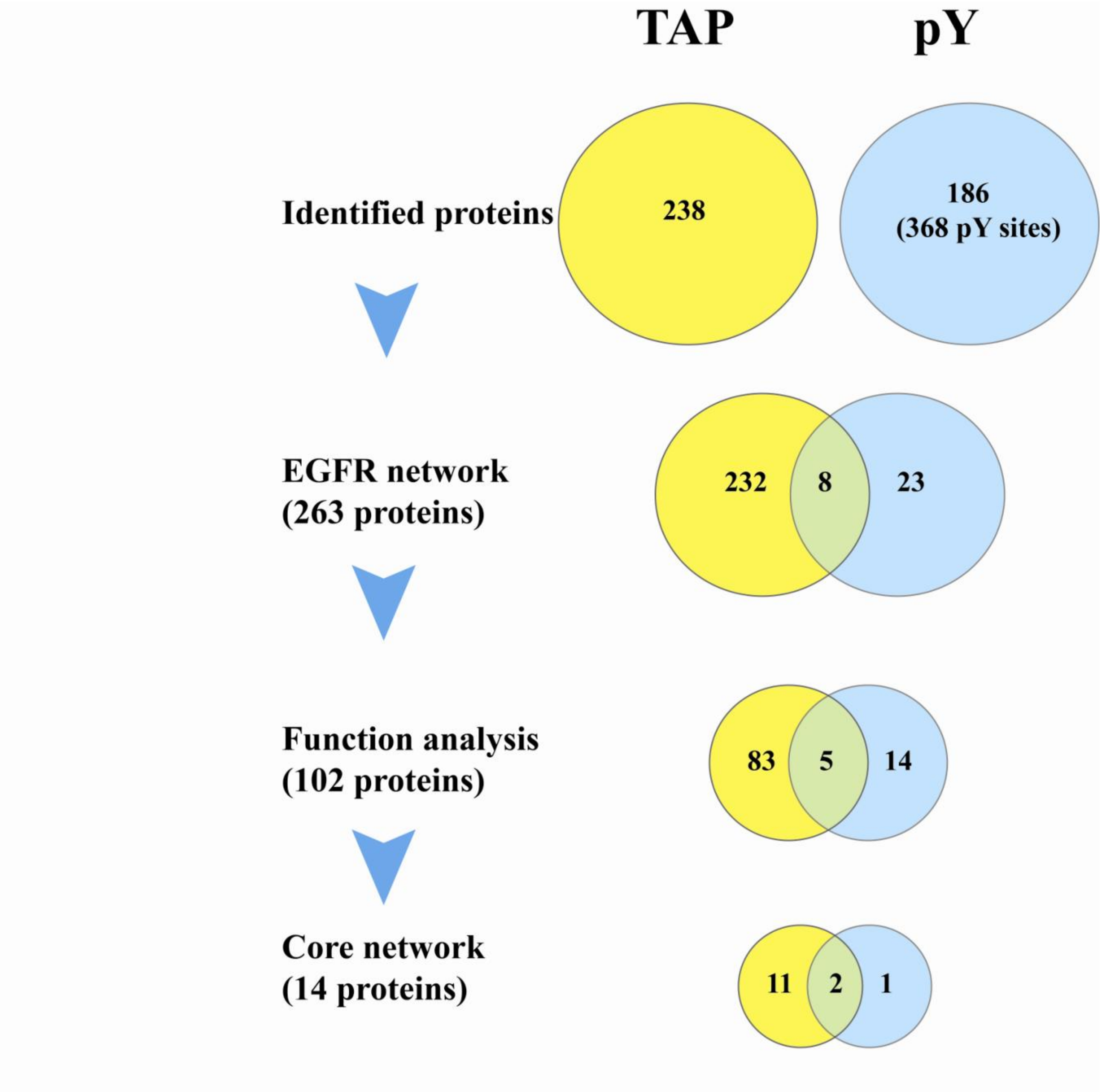

Supplement: Supplementary Information — Supplementary Table S1, Suppementary Figures S1-6 [file msb201361-s1.pdf]
